# Big little lies: a compendium and simulation of *p*-hacking strategies

**DOI:** 10.1098/rsos.220346

**Published:** 2023-02-08

**Authors:** Angelika M. Stefan, Felix D. Schönbrodt

**Affiliations:** ^1^ Department of Psychology, University of Amsterdam, Amsterdam, The Netherlands; ^2^ Department of Psychology, Universität der Bundeswehr München, München, Germany; ^3^ Department of Psychology, Ludwig-Maximilians-Universität München, Munchen, Germany

**Keywords:** questionable research practices, simulation, error rates, significance, false-positive rate, Shiny app, *p*-curve

## Abstract

In many research fields, the widespread use of questionable research practices has jeopardized the credibility of scientific results. One of the most prominent questionable research practices is *p*-hacking. Typically, *p*-hacking is defined as a compound of strategies targeted at rendering non-significant hypothesis testing results significant. However, a comprehensive overview of these *p*-hacking strategies is missing, and current meta-scientific research often ignores the heterogeneity of strategies. Here, we compile a list of 12 *p*-hacking strategies based on an extensive literature review, identify factors that control their level of severity, and demonstrate their impact on false-positive rates using simulation studies. We also use our simulation results to evaluate several approaches that have been proposed to mitigate the influence of questionable research practices. Our results show that investigating *p*-hacking at the level of strategies can provide a better understanding of the process of *p*-hacking, as well as a broader basis for developing effective countermeasures. By making our analyses available through a Shiny app and R package, we facilitate future meta-scientific research aimed at investigating the ramifications of *p*-hacking across multiple strategies, and we hope to start a broader discussion about different manifestations of *p*-hacking in practice.

## 1. Introduction

In an academic system that promotes a ‘publish or perish’ culture, researchers are incentivized to exploit degrees of freedom in their design, analysis and reporting practices to obtain publishable outcomes [1]. In many empirical research fields, the widespread use of such questionable research practices has damaged the credibility of research results [2–5]. Ranging in the grey area between good practice and outright scientific misconduct, questionable research practices are often difficult to detect, and researchers are often not fully aware of their consequences [6–8].

One of the most prominent questionable research practices is *p*-hacking [4,9]. Researchers engage in *p*-hacking in the context of frequentist hypothesis testing, where the *p*-value determines the test decision. If the *p*-value is below a certain threshold *α*, it is labelled ‘significant’, and the null hypothesis can be rejected. In this paper, we define *p*-hacking broadly as any measure that a researcher applies to render a previously non-significant *p*-value significant.

*p*-hacking was first described by De Groot [10] as a problem of multiple testing and selective reporting. The term ‘*p*-hacking’ appeared shortly after the onset of the replication crisis [9,11], and the practice has since been discussed as one of the driving factors of false-positive results in the social sciences and beyond [12–14]. Essentially, *p*-hacking exploits the problem of multiplicity, that is, *α*-error accumulation due to multiple testing [15]. Specifically, the probability to make at least one false-positive test decision increases as more hypothesis tests are conducted [16,17]. When researchers engage in *p*-hacking, they conduct multiple hypothesis tests without correcting for the *α*-error accumulation, and report only significant results from the group of tests. This practice dramatically increases the percentage of false-positive results in the published literature [18].

The current literature typically depicts *p*-hacking as an inherently atheoretical and incentive-driven procedure (e.g., [14,19,20]). When engaging in *p*-hacking, researchers are assumed to explore different data analysis options in a trial and error fashion, fishing for ‘publishable’ statistically significant findings with little regard to the theoretical underpinnings of their research. This iterative testing procedure only ends once the researcher has obtained their desired result or has run out of creativity or patience [21].

Fishing for publishable results is not confined to the frequentist hypothesis testing framework, but can occur in any situation where arbitrary qualitative thresholds on statistical quantities determine publication success [22]. However, in the literature, the term ‘*p*-hacking’ has been reserved for frequentist hypothesis testing where the *p*-value is the target of statistical fishing expeditions. Other terms were introduced to describe similar practices in other statistical frameworks, such as ‘*B*-hacking’ or ‘posterior hacking’ in Bayesian statistics [9,23]. In line with the existing literature, we therefore view *p*-hacking as a special case of a more general class of outcome fishing and data dredging practices.

It should be emphasized that not every researcher engaging in *p*-hacking is fully aware of its ramifications [6,22]. There are many degrees of freedom in statistical analyses, and usually, there is more than one right way through the proverbial garden of forking paths [24]. This arbitrariness constitutes an ideal breeding ground for biases and motivated reasoning that can provide researchers with subjectively convincing arguments to justify their analytic choices in hindsight. Therefore, *p*-hacking is not necessarily an intentional attempt at gaming the system, but can also be a product of human fallibility [25].

To mitigate *p*-hacking, it is of essence to raise awareness of degrees of freedom in the design, analysis, and reporting of statistical analyses that can be exploited for the purpose of rendering statistically non-significant results significant [26]. In fact, *p*-hacking is often defined by example as a vague compound of strategies, where each strategy utilizes a different aspect of analytical flexibility to push the *p*-value below the significance threshold. For example, Simonsohn *et al.* ([9], p. 670) write ‘a researcher may run a regression with and without outliers, with and without a covariate, with one and then another dependent variable, and then only report the significant analyses in the paper’. Other articles use different examples for *p*-hacking strategies, with a varying degree of detail and overlap between the listed strategies (e.g., [6,14]). Simulation studies that demonstrate the impact of *p*-hacking then usually focus on a small subset of these strategies, making conclusions in a pars-pro-toto manner (e.g., [1,12,18,27,28]). Thus, while many authors have warned researchers of dangers of certain *p*-hacking strategies, a comprehensive overview of these strategies that evaluates the severity of their consequences is currently missing.

We believe that a compilation and thorough description of *p*-hacking strategies and their ramifications can be beneficial for several reasons. First, it will enable researchers to identify possible sources for *p*-hacking more easily and to target them with appropriate measures. This is particularly true for *p*-hacking strategies that have been rarely mentioned in the literature, and might therefore still pass underneath the radar despite concerted efforts to mitigate questionable research practices. Second, a demonstration of the distinctive impact of a range of *p*-hacking strategies will be useful for future simulation studies that model *p*-hacking behaviour. We believe that simulation studies can be more realistic by incorporating a variety of *p*-hacking strategies instead of extrapolating from a few select strategies. Since the severity of *p*-hacking differs per strategy, it is also recommended that methods for *p*-hacking detection should be tested for their sensitivity based on a combination of different *p*-hacking strategies. For this purpose, it will be useful to have access to a compendium of computational descriptions of *p*-hacking strategies. Lastly, meta-scientific research will benefit from a better overview of *p*-hacking strategies, among others, to formulate more specific questions in surveys on questionable research practices. The past years have shown a surge of survey and interview based research on scientific practices, with a particular focus on the prevalence of misconduct and malpractice [4,5,29,30]. We believe that when assessing the prevalence of *p*-hacking in a field, it is beneficial to consider a wide range of strategies since different fields may vary in their susceptibility to different *p*-hacking strategies.

In this paper, we provide a closer look at various *p*-hacking strategies and investigate their distinctive impact on the credibility of research results. Based on extensive literature research, we compile an overview of 12 *p*-hacking strategies, and identify factors that control their level of severity. For each of the strategies, we provide a small simulation study that demonstrates the increase in false-positive findings as a result of applying the *p*-hacking strategy to a commonly used hypothesis test. Additionally, we illustrate through simulation how effects of different *p*-hacking strategies can accumulate when they are iteratively applied to the same dataset. To facilitate the re-use of our simulation environment for research and educational purposes, we also provide an R package and a *Shiny* web-application that can be used to explore additional simulation conditions. Finally, we use the results of our simulation studies to critically examine the usefulness of different methods that have been proposed to mitigate the detrimental effects of *p*-hacking.

We would like to note that throughout this paper we will frequently describe undesirable actions of researchers using phrases that suggest intention. This is purely for the purpose of better readability, and does not imply that all researchers engaging in *p*-hacking are knowingly deceptive. Although we believe that *p*-hacking is still common in many fields, we do not intend to make claims about the proportion of researchers engaging in *p*-hacking against their better knowledge. Moreover, we would like to stress that this paper should in no way be interpreted as an instruction manual for ‘successful’ *p*-hacking. We sincerely hope that our simulation results vividly illustrate the adverse effects of *p*-hacking and will therefore discourage readers from engaging in questionable research practices.

## 2. *p*-hacking and other questionable research practices

Before diving into the specifics of different *p*-hacking strategies, it is important to differentiate *p*-hacking from other questionable research practices. In the literature, *p*-hacking is often mentioned together with HARKing [31] and publication bias [32].

### 2.1. HARKing

HARKing is an acronym that stands for ‘hypothesizing after the results are known’. The term was coined by Kerr [31] and describes the practice of presenting *post hoc* hypotheses in the introduction of an academic paper as if they had been formulated before seeing the data. According to Mills [33], HARKing can be subsumed under opportunistic malpractices, since the researcher is indifferent with regard to the tested hypotheses, and bends the storyline of their paper to fit the data. By contrast, *p*-hacking would be described as procrustean practice, that is ‘making the data fit the hypothesis’.^1^ Here, a researcher is setting out to find proof for a hypothesis, and performs changes in the analysis pipeline to obtain the desired result. Thus, although *p*-hacking and HARKing both involve computing multiple statistical tests, the conceptual target hypothesis remains constant with *p*-hacking, whereas it may change considerably with HARKing.

The demarcation between *p*-hacking and HARKing is not always clear. For example, the practice of computing hypothesis tests with multiple dependent variables is often seen as an instance of *p*-hacking (e.g. [27,34]), but can also be viewed as HARKing, depending on the degree of conceptual difference between the measured variables. Similarly, re-calculating a hypothesis test in different subgroups (e.g. described in [35]) can also be conceptualized as HARKing or *p*-hacking, depending on whether a significant result is presented as an *a priori* moderation hypothesis, or as support for a more general subgroup-independent hypothesis that a researcher favours. In practice, extreme forms of *p*-hacking may often be considered HARKing, since a justification for uncommon analysis options may require additional *post hoc* hypothesizing. For this reason, as well as due to the fact that *p*-hacking and HARKing both increase false-positive rates, we will include borderline cases in our compendium of *p*-hacking strategies, and leave the choice of terminology to the reader.

### 2.2. Publication bias

The term *publication bias* describes a situation where the published scientific literature in a field is unrepresentative of the population of completed studies ([32], p. 1). Typically, publication bias is portrayed as being exerted by journal editors and reviewers who function as gatekeepers for scientific publication, and favour significant over non-significant results for publication [36,37]. However, there is evidence that publication bias can also be self-imposed by scientists who decide against submitting reports containing non-significant results [38,39].

Self-imposed publication bias bears many similarities to *p*-hacking, as both practices concern selective reporting of significant results. Some authors even consider selective reporting of studies based on statistical significance parts of *p*-hacking strategies [8]. Here, we limit our definition of *p*-hacking to measures applied to a single dataset. Additionally, we distinguish between **p*-hacking strategies* that researchers use to exploit the problem of multiplicity with the goal of obtaining significant results, and *reporting strategies* that researchers use to decide which test results they make public. Specifically, several authors have suggested that researchers may conduct analyses in batches (e.g. five analyses, each with a different outlier detection method), and then use a *p*-value dependent choice rule to decide which analysis to report (e.g. ‘report the smallest *p*-value’; [8,9]). Here, we decided to treat these reporting strategies independent of *p*-hacking strategies. Our rationale is that each *p*-hacking strategy (with the exception of optional stopping) can be combined with one of several different reporting strategies, but the reporting strategy does not influence the ‘success’ rate of *p*-hacking, that is, whether a non-significant result could be made significant. For example, if multiple outlier detection methods are tried out (see below), the success of the *p*-hacking attempt is only determined by whether *at least one* of the methods returns a significant *p*-value. If more than one significant result was obtained, the *p*-hacking attempt was successful, regardless of which *p*-value the researcher chooses to report.

## 3. A compendium of *p*-hacking strategies

In the following part of this paper, we will present 12 *p*-hacking strategies that we compiled based on an extensive literature review. For each strategy, we will provide a detailed description based on published literature as well as a small simulation study investigating the effects of the respective *p*-hacking strategy on the rate of false-positive results.

It is important to note that our list of *p*-hacking strategies should not be viewed as exhaustive. In the same way that it is impossible to map out all degrees of freedom that exist in all methods of data analysis in the quantitative sciences, it is an impossible endeavour to list all questionable research practices that misuse this flexibility. Fields that involve highly specialized data preprocessing pipelines, such as EEG or eye-tracking research, may also be subject to highly specialized forms of *p*-hacking that are not typically considered in the literature [40,41]. This may also be true for fields that employ highly sophisticated statistical analysis techniques, such as cognitive modelling [42].

In this paper, we will focus on *p*-hacking strategies that have been repeatedly mentioned in the statistical and meta-science literature. They are not field-specific and can be applied to a wide range of research designs and hypothesis testing scenarios. In our simulations, we will demonstrate their ramifications using the example of *t*-tests and (univariate) linear regressions, giving preference to the *t*-test whenever possible. We use these statistical testing procedures for two reasons: first, *t*-tests and regression analyses are among the most widely used hypothesis testing procedures in the social sciences and beyond [43,44]; second, they have typically been used in previous simulation studies to demonstrate the effects of *p*-hacking, so our simulation results can be directly compared to existing similar simulation studies (e.g. [8,9]). It is important to bear in mind that while the listed *p*-hacking strategies could also be applied to more complex statistical procedures, their effects on false-positive rates may differ for these analyses. Our simulation results should therefore only be interpreted with respect to the analyses used therein.

All of our simulations will be conducted assuming a significance threshold of *α* = 0.05, which corresponds to the conventional significance threshold in the social sciences [45]. We will simulate data under a true population effect size of zero. This implies that all statistically significant test results in the simulation are by definition false positive. Simulations of type I error rates will use 30 000 iterations of the Monte Carlo sampling procedure for each simulation condition, based on 10 000 iterations for each of three simulated reporting strategies. For each *p*-hacking strategy, we will simulate outcomes for sample sizes of *N* ∈ {30, 50, 100, 300}. For the *t*-test, these signify the sample size per group, for regression analyses the total sample size. These sample sizes are typical for the social sciences [46,47], and we vary them consistently for each *p*-hacking strategy to examine the robustness of *p*-hacking effects to different sample sizes. Additionally, we will investigate each *p*-hacking strategy under different levels of *p*-hacking aggressiveness, that is, under several variations of the *p*-hacking procedure that are under the control of the researcher and can influence the severity of *p*-hacking effects. Moreover, where necessary, we also investigate different data environments, that is, correlational relationships of the involved variables. Our goal is to showcase a plausible range of *p*-hacking effects for different levels of *p*-hacking aggressiveness and different data environments.

Our judgement of plausibility is based on the assumption that researchers do not automate *p*-hacking procedures (e.g. loop through a number of covariates using a script), that the research context imposes natural limits on the number of plausible analysis pipelines (e.g. subgroup effects that can be theoretically motivated *post hoc*), and that the researcher’s knowledge about different analysis pathways is limited (e.g. researchers are familiar with only a few outlier detection methods). However, in different research fields, different levels of *p*-hacking aggressiveness may be plausible. To make it simple for the reader to explore such settings in simulations, we provide an interactive *Shiny* app, as well as an R package (both available through GitHub via https://github.com/astefan1/phacking_compendium). Code to reproduce the simulations conducted in this paper can be found in an associated OSF repository https://osf.io/5nbkc/ as well as on Github. An overview table listing all 12 *p*-hacking strategies together with the reported simulation conditions can be found in our electronic supplementary material, online appendix on the OSF.

### 3.1. Selective reporting of the dependent variable

Selective reporting of significant results from a series of hypothesis tests with different dependent variables is one of the most frequently cited examples of *p*-hacking (e.g. [6,9,12,14,26,48,49]). There is substantive evidence that a large number of researchers have engaged in this questionable research practice. Specifically, with more than 60%, selective reporting of the dependent variable was the questionable research practice with the highest admission rate, and also received the highest defensibility rating in John *et al*.'s [4] survey on questionable research practices. Additionally, an analysis of Cochrane reviews found a major change in outcome variables compared to a previously submitted protocol for 46.8% of the publications [50], and a review of randomized trials in Denmark found that 50% of efficacy and 65% of harm outcomes per trial were incompletely reported [51]. This suggests that selective reporting of dependent variables is a common practice, even in fields with high standards for study preregistration.

Starting with Simmons *et al.*'s [7] seminal paper criticizing false-positive results in psychology, selective reporting of dependent variables has been a preferred target for simulations of *p*-hacking. In an exchange of papers discussing the potential of publication bias correction methods to account for *p*-hacking, Simonsohn *et al.* [34], Ulrich & Miller [8] and van Aert *et al.* [27] provided simulations of selective outcome reporting in *t*-tests with up to 32 dependent variables. Bishop & Thompson’s [52] simulation stressed the effect of correlation between dependent variables on the severity of *p*-hacking and introduced the term ‘ghost variables’ for dependent variables that are not reported in the main study. Bakker *et al.* [1] simulated selective reporting of the dependent variable in combination with other questionable research practices to illustrate how researchers game the scientific publication system. All above-mentioned simulations focused on comparisons between two groups, using independent-sample *t*-tests.

**Figure 1.**
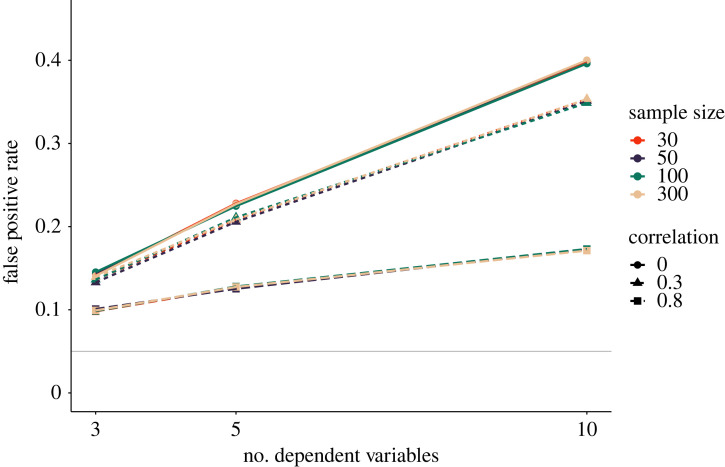
Impact of selective reporting of the dependent variable on false-positive rates in a *t*-test. Number of dependent variables indicates how many hypothesis tests were conducted (at maximum) to obtain a significant result. The solid grey line shows the nominal *α*-level of 5%.

Our simulation below provides an impression of the severity of the *p*-hacking strategy. We varied the number of dependent variables, *k* ∈ {3, 5, 10}, as well as the correlation between the dependent variables, *r* ∈ {0, 0.3, 0.8}, for *p*-hacking applied to a *t*-test. As shown in figure 1, false-positive rates increase up to roughly 40% for 10 uncorrelated dependent variables. Severity of *p*-hacking decreases with higher correlations and fewer dependent variables. Sample size does not appear to be a protective factor towards the *p*-hacking strategy.

### 3.2. Selective reporting of the independent variable

Selective reporting is not restricted to dependent variables. The rate of false-positive results can also be increased by selectively reporting significant hypothesis testing results for several independent variables [26,53,54]. In the literature, this *p*-hacking strategy is often characterized as comparing multiple experimental conditions to a control condition and selectively reporting results from significant comparisons [7,27,55,56]. Consequently, simulations of the *p*-hacking strategy have focused on the *t*-test where a control group is kept constant, and multiple experimental groups are compared to the control group [27,55]. However, selective reporting of the independent variable can also occur in the context of other statistical tests. For example, in regression analyses, selective changes to the predictor variable are equivalent to selective reporting of independent variables.

Interestingly, compared to selective reporting of the dependent variable, selective reporting of the independent variable is mentioned considerably less often in the literature and also seems to have a lower prevalence. In the survey on questionable research practices by John *et al.* [4], 27.7% of researchers admitted to ‘failing to report all of a study’s conditions’—roughly half of the admittance rate for selective reporting of the dependent variable. Franco and colleagues [56] reported a similar prevalence for survey experiments in political science based on empirical data. One explanation for the lower prevalence may be the increased effort of adding participants to experimental groups versus adding an additional observation for each participant.

**Figure 2.**
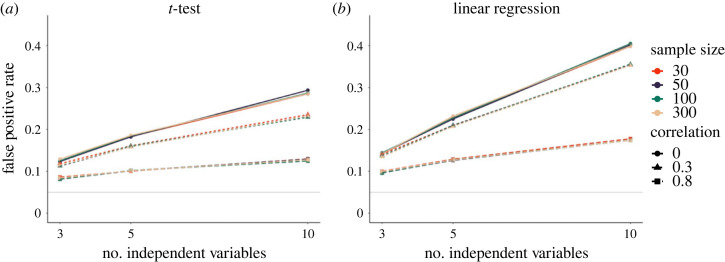
Impact of selective reporting of the independent variable on false-positive rates. Number of independent variables indicates how many hypothesis tests were conducted (at maximum) to obtain a significant result. The solid grey line shows the nominal *α*-level of 5%. (*a*) False-positive rates for the *t*-test. (*b*) False-positive rates for a univariate regression.

In line with previous research, we first used a *t*-test to illustrate the severity of the *p*-hacking strategy in our simulations. We varied the number of experimental conditions (i.e. independent variables) with *k* ∈ {3, 5, 10}, and the correlation between them with *r* ∈ {0, 0.3, 0.8}. To simulate selective reporting of the independent variable, we compared each experimental group to a fixed control group. In a second step, we applied the *p*-hacking strategy to univariate linear regression with *k* ∈ {3, 5, 10} candidate predictor variables that were correlated at *r* ∈ {0, 0.3, 0.8}. As shown in figure 2, *p*-hacking severity in terms of false-positive results increases with an increasing number of independent variables, as well as with a decreasing correlation between them. Sample size is not a protective factor towards the *p*-hacking strategy. Notably, the *p*-hacking strategy has more severe effects in regression (cf. figure 2*b*) than in *t*-tests (cf. figure 2*a*). This is due to the fact that in the experimental-control group scenario, only half of the data in the independent variable (the experimental group) is exchanged, whereas in the regression example, the entire predictor variable is exchanged. For regression, the effects of *p*-hacking by selectively reporting the independent variable are therefore more comparable to selective reporting of the dependent variable, while the effects for *t*-tests are somewhat lower.

### 3.3. Optional stopping

Optional stopping, also often referred to as ‘data peeking’, occurs when a researcher repeatedly computes a hypothesis test as data accumulate, and stops collecting data once a significant result has been obtained or a maximum sample size has been reached [16]. It is one of the most frequently mentioned *p*-hacking strategies in the literature (e.g. [6,8,12,14,57–59]), and has an admittance rate of 15.6% in John *et al*.'s [4] survey. Optional stopping differs from other *p*-hacking strategies in that it actively influences the data collection process. Whereas other *p*-hacking strategies assume that the researcher selectively analyses variables or observations in an existing dataset, optional stopping leads to an expansion of an initial dataset, while data preprocessing and analysis pipelines remain constant.

The severity of optional stopping depends on the number of peeks a researcher takes at the data, as well as on the number of observations that are added between two peeks. Theoretically, if a researcher had infinite resources, they would be guaranteed to obtain a significant result eventually [16]. However, in practice, researchers are subject to constraints of time, money, and patience, that will influence their sampling strategy [60]. Existing simulation studies suggest that there is little agreement on how often researchers would be willing to peek at their data. Some simulation studies assumed that researchers peek at their data only a few times. For example, Simmons *et al.* [7] and Simonsohn *et al.* [34] simulated a single increase in sample size from *N* = 20 to *N* = 30 if the initial result was not significant. Bakker *et al.* [1] also simulated an increase of 10 participants, but combined optional stopping with two other *p*-hacking strategies in their simulations. Hartgerink *et al.* [61] simulated optional stopping as a process with three stages, where sample size was increased by one-third of the initial sample size in each stage. By contrast, simulations by Lakens [55] and Armitage *et al.* [16] assumed that researchers check the results of the hypothesis testing procedure after every participant, up to a maximum sample size of *N* = 100 and *N* = 1000, respectively.

Here, we conducted two simulation studies to investigate the effects of optional stopping. In the first set of simulations, we fixed the initial sample size to *N*_min_ = 5, and varied the maximum sample size to *N*_max_ ∈ {30, 50, 100, 300} (consistent with the sample sizes simulated for other *p*-hacking strategies). We also varied the step size, that is, the number of observations collected in one batch, with *k* ∈ {1, 5, 10, 50}. If the step size was larger than the difference between minimum and maximum sample size, we simulated two peeks: one at the minimum and one at the maximum sample size. All conditions were simulated for a *t*-test, and observations were added to both groups at a time. In a second set of simulations, we varied the minimum sample size instead with *N*_min_ ∈ {5, 30, 50, 100}. The step sizes considered were the same as in the first set of simulations.

**Figure 3.**
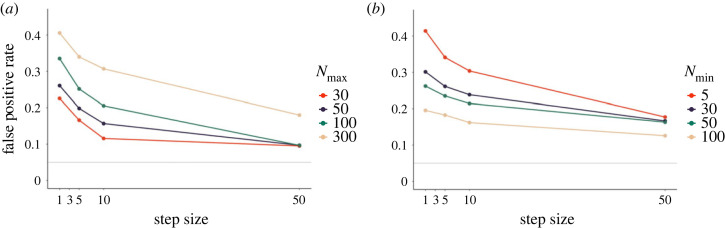
Impact of optional stopping on false-positive rates in a *t*-test. The solid grey line shows the nominal *α*-level of 5%. (*a*) False-positive rates dependent on step size and maximum sample size. Minimum sample size is fixed to *N* = 5. Step size indicates how many participants were added in a batch. (*b*) False-positive rates dependent on step size and minimum sample size. Maximum sample size is fixed to *N* = 300.

Figure 3 depicts the results of the two sets of simulations. Panel (*a*) shows the simulations where *N*_max_ was varied, panel (*b*) shows the simulations where *N*_min_ was varied. It becomes clear from (*a*) that a higher maximum sample size and a smaller step size increase the risk of obtaining a false-positive result. Similarly, Panel (*b*) shows that a smaller minimum sample size and a smaller step size lead to higher rates of false-positive results. This means that more interim peeks at the results lead to higher false-positive rates. If a researcher decides to check the results after every single participant, false-positive rates are especially elevated, even if the maximum sample size is small or the minimum sample size is large. This shows that optional stopping can have a large impact even if resource constraints prohibit researchers from collecting large sample sizes or academic journals impose minimum sample size requirements.

### 3.4. Outlier exclusion

One of the most common examples of *p*-hacking is ‘data trimming’, the selective exclusion of data points (e.g. [6,8,9,14,26,58,59,62]). Typically, the justification provided for excluding these data points is that they can be considered outliers, that is, they are markedly different from other data values [63]. *p*-hacking based on outlier exclusion exploits the ambiguity surrounding the definition of outlier values, as well as the methodological flexibility surrounding outlier handling [63,64]. Over time, many outlier detection methods have been developed, ranging from visual inspection of data plots to investigation of distance measures or variable influence metrics. In a literature review, Aguinis *et al.* [63] listed 39 different outlier identification techniques, each of them bearing the potential for additional analytical flexibility through, for example, different cut-off specifications.

Empirical evidence suggests that many authors are vague when it comes to reporting outlier detection methods, or even fail to mention outlier exclusion altogether. Bakker & Wicherts [65] showed that discrepancies in degrees of freedom and sample size were common in articles that did not report outlier exclusions, suggesting a failure to report data exclusion or missingness. Other literature reviews lament a general vagueness in reporting of outlier handling strategies, for example, it is often unclear whether cut-off values were selected in advance [63,64]. Deciding to ‘exclude data points after looking at the impact of doing so on the results’ was also a questionable research strategy with a high admission rate in John *et al*.'s [4] survey, as well as in a survey among criminology researchers by Chin *et al.* [66], with 38.2% and 24%, respectively.

Many modern outlier detection techniques are geared towards regression analyses. Therefore, it is interesting to see that simulations of *p*-hacking through outlier exclusion have so far only targeted *t*-tests. Simonsohn *et al.* [34] simulated a scenario where researchers selectively drop values that are at least two standard deviations away from the group mean—either for one of the groups or for both. Bakker *et al.* [1] used the same definition of outliers in their simulation of a combined *p*-hacking strategy. In a larger simulation study on the impact of outlier removal on hypothesis testing results, Bakker & Wicherts [65] defined ‘subjective’ removal of outliers as choosing a threshold of 2, 2.5, or 3 standard deviations, depending on the statistical significance of the hypothesis test. They found increased type I error rates of up to 45%, most prominently when the data were simulated from a skewed population distribution.

In our simulation of *p*-hacking through outlier exclusion, we investigate the consequences on type I error rates based on a regression analysis. We use a regression analysis because this allows us to incorporate a broader spectrum of outlier detection techniques. The underlying idea is that if researchers are knowledgeable about outlier identification methods, they can exploit more degrees of freedom in a regression analysis than in a *t*-test. Our simulation comprises a total of 12 outlier detection techniques. For single-variable detection techniques, our simulation method is similar to Simonsohn *et al.* [34]. We determine outliers both in the predictor (*x*) and in the outcome variable (*y*), and successively delete observations where *x* is an outlier, *y* is an outlier, or *x* *and* *y* are outliers. For each outlier detection technique, we additionally simulate subjective threshold setting similar to Bakker & Wicherts [65]. For example, for the standard deviation method, we assume that a researcher would start with a (lenient) threshold of two standard deviations and increase the threshold successively by 0.5 standard deviations until no more extreme data points are in the specified region. Note that we simulate outlier removal as a single-step procedure [67]. That is, outlier detection methods are applied only once to a dataset, and detected outliers are removed—the reduced dataset is not re-evaluated with regard to potential outliers. In our simulation study, we assume that researchers differ in their knowledge about outlier detection techniques. We implemented this by drawing *k* ∈ {3, 5, 12} techniques at random from the total number of outlier detection techniques in each iteration of the simulation. With the simulation functions in the R package and *Shiny* app, readers can explore the effect of *p*-hacking with specific outlier detection methods, similar to simulations by Bakker & Wicherts [65] and Simonsohn *et al.* [34]. Our simulation study assumes that both variables involved in the regression analysis are normally distributed. Therefore, the observed increase in false-positive rates can be attributed only to *p*-hacking and not to a violation of statistical assumptions. We provide an overview of all outlier detection techniques and a detailed description of our simulation method for each technique in electronic supplementary material, table A2 of our online appendix.

**Figure 4.**
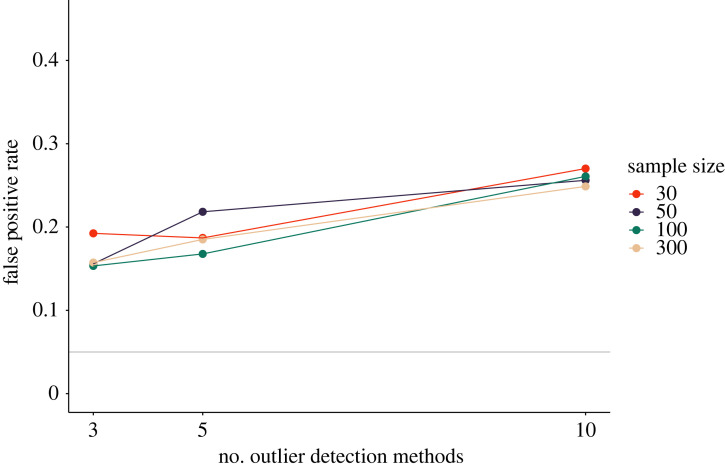
Impact of outlier exclusion on false-positive rates in a univariate regression. The x-axis shows the number of outlier detection methods that were applied. For each outlier detection method, at least three hypothesis tests were conducted (excluding outliers in *x*, *y*, and *x* and *y*). The solid grey line shows the nominal *α*-level of 5%.

Figure 4 shows the results of our simulation study. If all 12 simulated outlier detection strategies are applied successively, the probability of obtaining a significant result rises up to almost 30%, but even for few outlier detection methods, type I error rates are high. Sample size appears to interact with the number of outlier detection methods that are applied. When few outlier detection methods are applied, the type I error rate differs visibly for different sample sizes, but if all 12 candidate outlier detection methods in our set are applied, sample size plays almost no more role. This may be explained by the differential sensitivity of different outlier detection methods under different sample sizes and their probability of being in the selected set.

### 3.5. Controlling for covariates

The importance of controlling for confounding variables has often been pointed out in the literature (e.g. [68]). However, controlling for covariates also constitutes a major source of analytic flexibility [9,31]. Many authors have cautioned that this analytic flexibility might be exploited by researchers who decide to include covariates in their model based on statistical significance (e.g. [8,14,26,34,49,69]).

There is substantive empirical evidence for *p*-hacking using covariate selection. In a comparison of doctoral dissertations and published journal articles describing the same studies, O’Boyle *et al.* [59] found that several authors had included additional boundary conditions in their hypotheses in the journal article that led to an increase in significant results (e.g. ‘X relates to Y [if Z is controlled for]’). Additionally, Simonsohn *et al.* [9] compared articles reporting analyses exclusively with a covariate to articles reporting analyses with and without the covariate. They found a left-skewed *p*-curve in the first case and a right-skewed *p*-curve in the latter, indicating that articles reporting analyses only with covariates might have hidden additional non-significant results. Finally, in a survey by Chin *et al.* [66], 32% of respondents admitted to dropping covariates selectively based on *p*-values.

Existing simulation studies of *p*-hacking with covariates either focused on regression analyses in observational data, where covariates are introduced as additional predictors [12,70], or on ANCOVAs testing whether the means of an experimental and control group differ after controlling for covariates [7]. Here, we take a similar approach to Simmons *et al.* [7] by extending a *t*-test with continuous covariates. We varied the number of covariates with *k* ∈ {3, 5, 10}, as well as the correlation between the covariates, *r*_*Z*_ ∈ {0, 0.3, 0.8} and the correlation between the covariates and the dependent variable, *r*_*ZY*_ ∈ {0, 0.3}. The *p*-hacking strategy was implemented as follows: first, the test was computed without covariates; then, all covariates were added separately to the model (i.e. $Y∼X+Z_{j}\;\mathrm{for}\;j\in 1,\ldots ,k$); and in a third step, the covariates were added sequentially to a model in decreasing order according to their correlation with the dependent variable in the sample (i.e. *Y* ∼ *X* + *Z*_1_ + · · · + *Z*_*k*_). For example, for three covariates, this simulated *p*-hacking strategy leads to five tests that are computed in addition to the original hypothesis test. We decided not to compute models with all combinations of covariates, as this would lead to a combinatorial explosion that would require researchers to use automation for exploring all analysis pathways.

**Figure 5.**
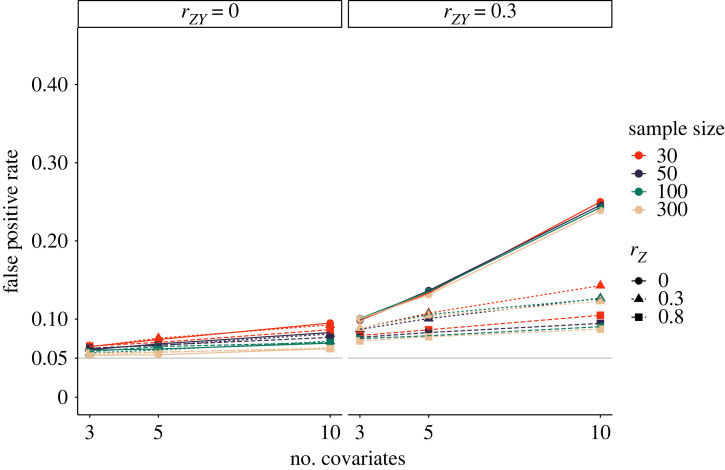
Impact of controlling for covariates in a *t*-test to fish for significant results. Plot shows false-positive rates depend on the number of covariates, correlation between covariates (*r*_*Z*_), correlation between covariates and dependent variable (*r*_*ZY*_), and sample size. The solid grey line shows the nominal *α*-level of 5%.

Figure 5 shows that the correlation between the covariates and the dependent variable has a strong effect on the impact of *p*-hacking on false-positive rates. *p*-hacking effects are stronger if the covariates correlate with the dependent variable. Specifically, both the number of covariates and the correlation between covariates only show a strong effect on false positive rates in the right panel of the figure. Additionally, sample size appears to be a slightly protective factor, in that false-positive rates for *N* = 30 are somewhat higher than for larger sample sizes.

### 3.6. Scale redefinition

In the social sciences, constructs are often measured indirectly through questionnaires or behavioural tasks. Researchers constantly develop new measurement tools and scales, and even for existing measures, there is often a high degree of flexibility in the computation of construct scores [71,72]. There are growing concerns that sub-standard measurement methods might undermine the trustworthiness of research results, culminating in warnings about a measurement crisis [73]. Interestingly, despite these concerns about measurement quality and flexibility, tinkering with measurement scales has been relatively rarely mentioned explicitly as a *p*-hacking strategy [8,59,70].

Existing research indicates that scale redefinition may have a substantive impact on statistical significance. For example, Elson *et al.* [71] investigated different analysis procedures for measuring aggressive behaviour with the Competitive Reaction Time Task and found large differences in *p*-values and effect sizes if different scoring rules were used. In a simulation study, Ulrich & Miller [8] investigated the opportunistic computing of composite scores as a *p*-hacking strategy. They assumed that researchers might compute composite scores from a number of correlated dependent variables by merging the variables that individually yield the smallest *p*-values, and found strongly right-skewed *p*-curves as a result. Ingre [70] simulated the use of different variations of scales for two predictors in five regression models, and observed false-positive rates of up to 97% based on the exploitation of the resulting multiplicity.

In contrast to Ulrich & Miller [8], we believe that the most common case of *p*-hacking based on scale redefinition might not be the haphazard computation of composite scores from multiple outcome variables, but the deletion of deviating items from measurement scales targeted at increasing internal consistency. This notion is supported by the fact that *SPSS* [74], a statistical software package commonly used in the social sciences, offers the popular option to automatically recalculate reliability coefficients for each item if the item was deleted from the score. We believe that this option encourages researchers to recompute their analyses with scales that are redefined to exclude seemingly ill-fitting items.

In our simulations, we implemented the resulting *p*-hacking strategy in the context of a regression analysis. We opted for a regression analysis for practical reasons to clearly differentiate the simulation from strategy 1 (selective reporting of the dependent variable). In our simulation, the *p*-value is first computed with the mean score of a scale as a predictor. Then, *m* items are successively removed based on their influence on Cronbach’s alpha. At each step, the hypothesis test is recomputed both with the reduced scale and with the deleted item as a predictor (the latter could be justified *post hoc* by the notion that the excluded item measured a different construct than the rest of the scale). We varied the number of original items with *k* ∈ {5, 10}, the correlation between the items, *r* ∈ {0.3, 0.7}, the maximum number of items that were deleted from the scale *m* ∈ {1, 3, 7}, and the sample size. Notably, our simulation strategy optimizes both the internal consistency of the scale and the *p*-value. Technically, it could therefore be considered a combination of ‘measurement hacking’ and *p*-hacking. However, given the strong incentives for high internal consistency, it is difficult to imagine a ‘pure’ *p*-hacking strategy.

**Figure 6.**
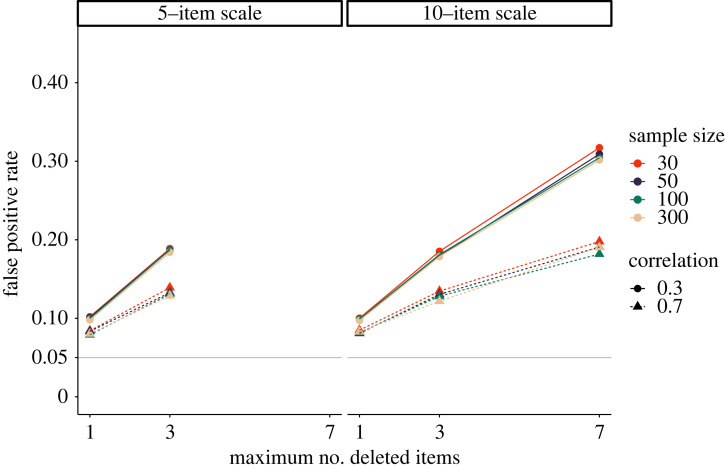
Impact of scale redefinition on false-positive rates. The number of computed hypothesis tests increases with the number of items that are excluded from the scale. The solid grey line shows the nominal *α*-level of 5%.

Figure 6 shows the results of our simulations. False-positive rates are higher if the correlation between the items on the scale is small. The number items in the scale only influence the severity of the *p*-hacking strategy insofar as more items can be deleted from longer scales, leading to a larger number of hidden hypothesis tests. False-positive rates increase drastically as more items are removed.

### 3.7. Variable transformation

Opportunistic variable transformation is closely related to the *p*-hacking strategy of scale redefinition. Transforming a variable, for example by computing its logarithm or reciprocal, changes the underlying measurement scale. However, for variable transformation, the measured variable does not need to be computed as a composite score.

For linear models, variable transformations of the dependent variable are often recommended to meet the model’s normality assumption [75]. Notably, the normality assumption is often misunderstood as referring to the normality of the outcome variable or predictors, but in fact refers to the normal distributions of residuals [76]. In practice, variable transformations directed at restoring normality may therefore often be statistically unsubstantiated. However, variable transformations are not always theoretically unjustified. For example, transformations of the independent variable in regression models are also sometimes recommended for the sake of working with a simple functional form in transformed variables, rather than a more complicated one on the original scale [77].

Opportunistic variable transformation has been identified by several sources as a *p*-hacking strategy (e.g. [6,62,69]). For example, Wicherts *et al.* [26] mention variable transformations conducted to approach normality as a degree of freedom in the analysis phase, and Wagenmakers *et al.* [20] criticize Bem’s [78] notorious ‘feeling the future’ study for exploiting arbitrary transformations of response times. However, so far, we have not found simulation studies investigating opportunistic variable transformation in the context of *p*-hacking.

In our simulation of variable transformation as a *p*-hacking strategy, we performed a regression analysis and used a logarithmic, reciprocal, and square root transformation on the dependent variable, the predictor variable, or both. We decided to use a regression analysis to simulate this *p*-hacking strategy, such that the same transformations could be performed on the outcome and predictor variable. Since a violation of the normality assumption is arguably the most common justification provided for variable transformation, we conducted the simulation with and without an initial normality check. In the normality-check condition, variables were transformed only if a Shapiro–Wilk test for normality of residuals was significant. Both variables in the simulation were drawn from a normal distribution, so any transformation of the outcome variable led to a violation of the normality assumption of the regression analysis. Our online appendix contains additional simulation results pertaining to the normality of residuals after transformation.

**Figure 7.**
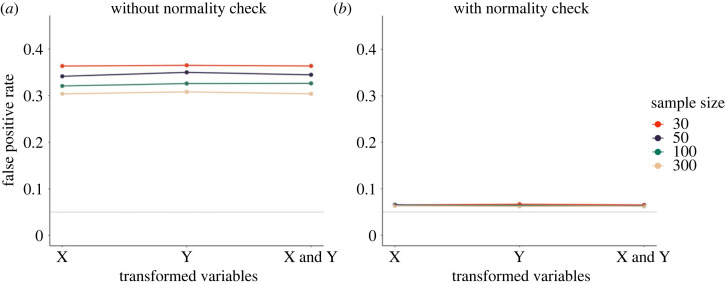
Opportunistic variable transformation as a *p*-hacking strategy. The plot shows false-positive rates when the predictor (X), the dependent variable (Y) or both were transformed using a logarithmic, reciprocal, and square root transformation. (*a*) False-positive rates if transformations are unconditionally applied. (*b*) False-positive rates if variable transformations are applied conditional on a significant normality test of residuals. The solid grey line shows the nominal *α*-level of 5%.

Figure 7 shows the results of the simulation. As can be seen in *a*, without the normality check, the rate of false-positive results increases to more than 25% for all three transformation conditions which can be viewed as a substantive overall increase. Interestingly, it seems to be inconsequential whether only the predictor or the outcome variable, or both, were transformed. The results indicate that sample size is a slightly protective factor, with a decrease of roughly five percentage points in false-positive rates between *N* = 30 and *N* = 300. Figure 7*b* shows that if the transformations are conducted conditional on a significant normality check, false-positive rates are substantially lower. The reason is that a false-positive result can only occur if the test for normality yields a false-positive result and the ensuing *p*-hacking through variable transformations is ‘effective’.

### 3.8. Discretizing variables

In statistical tests reported in published research, inherently continuous variables are often discretized into categories. For example, political orientation may be split into Democrat-leaning and Republican-leaning, participants may be split into old and young according to their age, or the duration of risk exposure may be split into long and short [22,70]. The selection of cut-off values is at the researchers’ discretion and constitutes a major source of analytic flexibility. Several authors have warned that this analytic flexibility could be misused for *p*-hacking (e.g. [22,33,48,70]).

Ingre [70] demonstrated the effect of discretization in a *p*-hacking simulation study based on research on night work on breast cancer in women. He adjusted for age using 10 different specifications of the age variable, from continuous, to quantile splits, to 5-year chunks with different reference ages. Additionally, he operationalized exposure to night work as a dichotomous variable, using eight different splitting mechanisms of a continuous predictor indicating the number of years worked in night shifts. Despite the large number of hypothesis tests, the results indicated only a small increase in false-positive rate.

**Figure 8.**
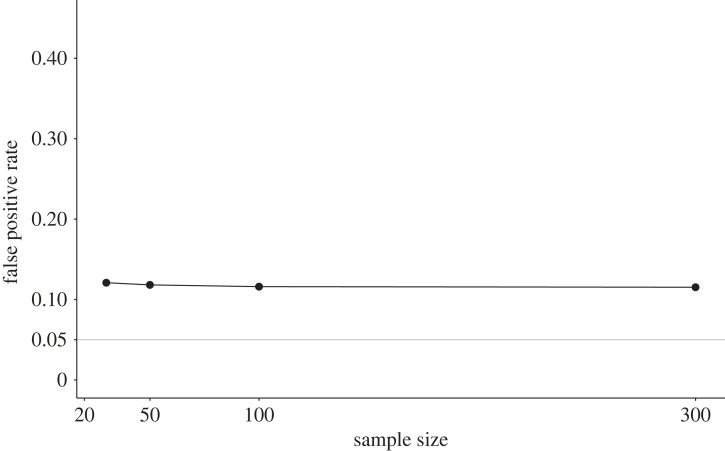
False-positive rates following the selection of one out of four predictor discretization methods based on *p*-value significance. The solid grey line shows the nominal *α*-level of 5%.

In our simulation, we applied three mechanisms to discretize a continuous variable: we used a median split, resulting in two groups, a tertile split, resulting in three groups, and a cut-the-middle split, where the middle category is removed after a tertile split to emulate a comparison of extreme groups. With regard to statistical testing procedures, this results in a regression analysis (for the continuous predictor), two *t*-tests (for the median split and the cut the middle strategy), and an ANOVA (for the tertile split) that are conducted to determine the relationship between two variables. Figure 8 shows that independent of sample size, false-positive rates are elevated by approximately five percentage points compared to the original *α*-level.

### 3.9. Exploiting alternative hypothesis tests

A single conceptual hypothesis can often be represented by multiple statistical models. Particularly when dealing with complex models, researchers typically have much freedom in adding or relaxing auxiliary assumptions, or changing the parametrization of the model [79]. This flexibility translates to model comparisons and hypothesis tests where multiple hypotheses are involved. Even for run-off-the-mill hypothesis tests, there usually exist several alternative options that restrict or relax distributional assumptions. Since these alternative hypothesis testing options do not always agree in their test decisions, they may be exploited in the context of *p*-hacking. For example, in her statement on *p*-hacking in power pose research, Dana Carney [80] reported having applied both a Pearson chi-squared and a likelihood ratio test, and selectively reporting only the significant test.

The practice of exploring different statistical modelling and hypothesis testing options has been mentioned several times as a *p*-hacking strategy in the literature (e.g. [6,69]). Often, it is presented alongside other analytical options, such as subgroup analyses, controlling for covariates, or choosing dependent variables [26,70]. Here, we chose to separate it from these strategies because using an alternative statistical hypothesis test does not necessitate adding, removing, or changing any of the involved variables.

We demonstrate the consequences of exploiting alternative hypothesis tests by simulating a scenario where a researcher switches between parametric and non-parametric versions of the same test. We believe that this scenario is particularly interesting to investigate since many statistical textbooks present these tests as epistemically equivalent and recommend a data-driven approach based on assumption checking for choosing between parametric and non-parametric tests (e.g. [81]). This legitimization of data-driven test selection could lead to a perceived vindication of *p*-hacking for this scenario in practice.

**Figure 9.**
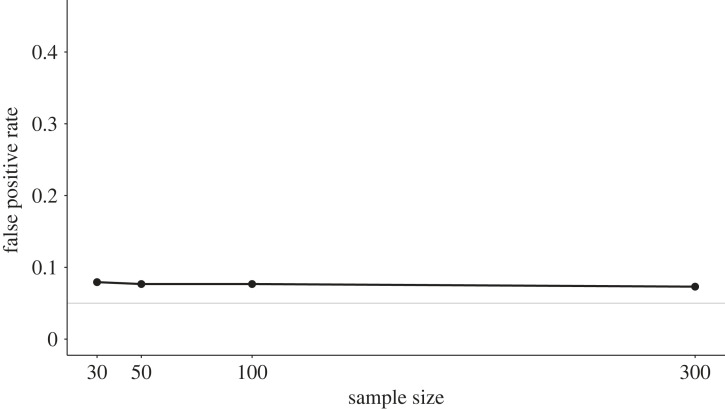
False-positive rates in a *t*-test after *p*-hacking using alternative non-parametric hypothesis tests. The solid grey line shows the nominal *α*-level of 5%.

In our simulation study, we implemented the *p*-hacking strategy by conducting an independent-samples *t*-test, a Welch test, a Wilcoxon test and a Yuen test with four different trimming parameters, *c* ∈ {0.1, 0.15, 0.2, 0.25}, on the same dataset [67]. Data were sampled from normal distributions with equal variances, such that the assumptions of the *t*-test were fulfilled in the population. The simulation results are displayed in figure 9. For all sample sizes, the false-positive rate lies at approximately 7.5%, and is therefore slightly increased compared to the nominal *α* level. While this *p*-hacking effect can be considered fairly small, it is important to note that larger effects can be expected for non-normal data or more complex models with more degrees of freedom.

### 3.10. Favourable imputation

In empirical research, missing data are often unavoidable. There are many possible options for dealing with missing data, the most fundamental decision being whether missing data should be deleted or replaced by plausible values—a technique also known as *imputation*. If a researcher decides to impute missing data, they can choose from a plethora of imputation methods, all leading to slightly different replacement values that can influence the results of statistical hypothesis tests [82].

Failing to fully disclose the handling of missing variables and the method used for imputation is considered a questionable research practice [83], and deciding on a favourable imputation method based on statistical significance constitutes a *p*-hacking strategy [26,84]. Meta-scientific evidence suggests that authors rarely provide a satisfactory justification for their chosen method for handling missing values [85,86]. Surveys on ethical research conduct among communication scientists and criminologists indicate that slightly less than 10% of researchers have hidden imputation methods from research reports [66,83].

We simulate *p*-hacking through favourable imputation using a total of 10 different methods for handling missing variables. A full list of imputation methods used in our simulation can be found in table A3 of our online appendix. Similar to our simulation of *p*-hacking through outlier exclusion, we assume that researchers are typically familiar with only a limited number of imputation methods. For this reason, we draw a random sample of *k* ∈ {3, 5, 10} techniques from the total number of imputation methods in each iteration. Additionally, we vary the proportion of data missing completely at random as *ρ* ∈ {0.05, 0.2}. Our simulation is based on a regression analysis since many modern imputation methods are designed for regression models [87]. Readers interested in exploring the effect of *p*-hacking with specific imputation methods can consult our R package and *Shiny* app.

**Figure 10.**
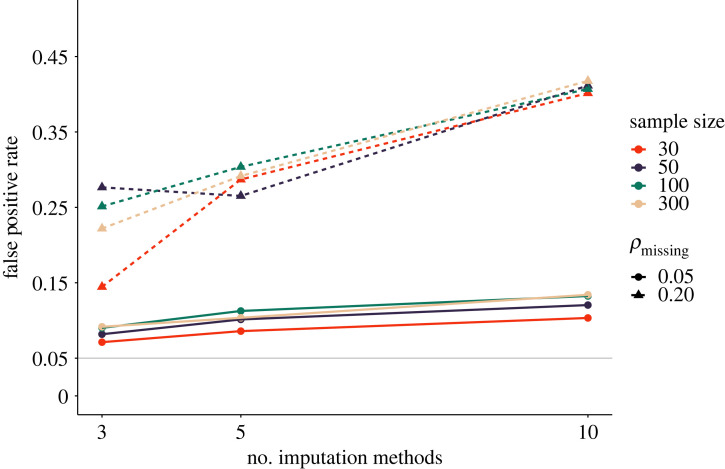
False-positive rates following the favourable imputation of missing values. *ρ*_missing_ indicates the proportion of missing values in the sample. The solid grey line shows the nominal *α*-level of 5%.

As shown in figure 10, the proportion of missing data has a substantive influence on the *p*-hacking severity. This is likely due to the fact that imputation methods judge the plausibility of parameter values based on trends in the data, and replacing large quantities of missing data with trend-conform values can make weak trends appear stronger. Interestingly, the number of imputation methods hardly plays a role if the proportion of missing values is small. However, even for small quantities of missing data, false-positive rates were slightly elevated under the influence of *p*-hacking.

### 3.11. Subgroup analyses

In social science studies, there are a multitude of potential reasons for restricting the sample to certain groups of participants. For example, participants may be excluded for failing attention checks, having missing values on core variables, or giving answers indicating that their data may be contaminated (e.g. being colour-blind in a Stroop task). Additionally, researchers may decide to include only certain subgroups in their sample, for example, elderly people, voters of a certain party, or people diagnosed with a certain disorder. Ideally, these decisions are determined *a priori* and justified by theory. However, there is anecdotal evidence that researchers frequently re-adjust their inclusion criteria after seeing the data, and only publish analyses based on a subgroup of observations. In meta-scientific articles, researchers have often noted that the same research teams used different participant inclusion rules for testing closely related hypotheses (e.g. [20,22,70]). In their comparison of Cochrane reviews to their published protocols, Silagy *et al.* [50] found that 34% of the reviews had made major changes to participant inclusion criteria. If inclusion criteria are changed based on the statistical significance of core hypothesis tests and the additional tests remain unreported, this practice constitutes a form of *p*-hacking [35,48,70].

Here, we simulate this *p*-hacking strategy using a *t*-test and *k* ∈ {1, 3, 5} binary grouping variables. Group membership is determined at chance for every participant, such that group sizes can differ. We believe that unequal group sizes are realistic in practice since participant recruitment would usually not balance out incidental grouping variables. We assume that the *t*-test is originally conducted on the whole sample and subsequently on both subgroups of a grouping variable. This mimics a scenario where researchers would claim the existence of an effect in a certain subgroup, for example women or older adults, if a significant result was found.

**Figure 11.**
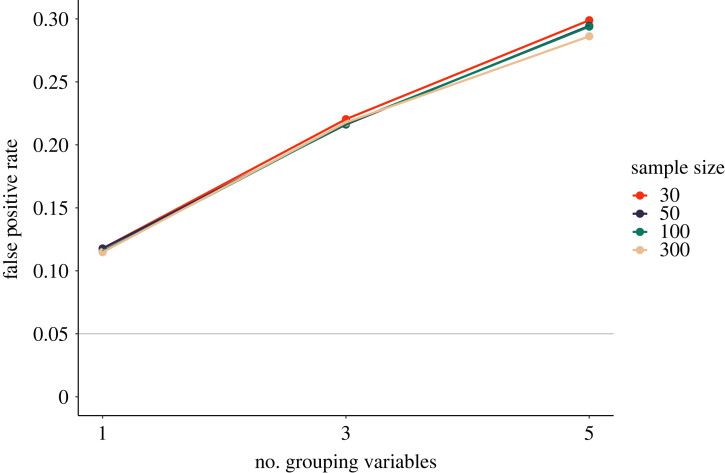
False-positive rates following subgroup analyses, assuming that a *t*-test is reconducted in both subgroups of one or more binary grouping variables. The solid grey line shows the nominal *α*-level of 5%.

Figure 11 shows the simulation results. The rate of false-positive results starts at slightly above 10% if only one grouping variable is exploited, and rises quickly as the number of grouping variables increases. False-positive rates are virtually independent of sample size, showing that larger sample sizes are not a protective factor against this *p*-hacking strategy.

### 3.12. Incorrect rounding

In a research environment where rewards are coupled to significant findings, obtaining a *p*-value of 0.0499 or 0.0501 can become a difference between success or failure. Therefore, if *p*-values are close to a significance threshold, researchers may be tempted to round them down and claim a significant finding. Several authors have recognized this as a *p*-hacking strategy (e.g. [14,61,88]).

Incorrect rounding differs from other *p*-hacking strategies in that it is possible to find direct evidence for it in the literature by recalculating *p*-values from test statistics. For example, Leggett *et al.* [88] extracted *p*-values from psychology literature between 1965 and 2005, and found that 36 out of 93 *p*-values reported as exactly *p*=0.05 were in fact larger. Hartgerink *et al.* [61] conducted a similar analysis on a sample of 2470 *p*-values reported as exactly *p*=0.05 and concluded that 67.45% of the *p*-values were incorrectly rounded down towards significance. Of course, these results are contingent on the assumption that test statistics were reported correctly. However, if both test statistic and *p*-value are intentionally misreported, it should be questioned if this should still be considered a questionable research practice or if it should be counted as outright fraud. In John *et al.*'s [4] survey, 22% of respondents admitted to ‘rounding off’ *p*-values. This indicates that although opportunity is scarce (after all, a *p*-value needs to fall within a narrow rounding margin), a considerable number of researchers engage in this *p*-hacking strategy.

Since the distribution of *p*-values under the null hypothesis is known, it is possible to determine the impact of incorrect rounding on the rate of false-positive results analytically. The effect depends on the rounding level, that is, the largest *p*-value that a researcher would be willing to round down to statistical significance. For example, if the nominal *α*-level is 0.05, researchers might round down values smaller than 0.051 or 0.06. Consequently, the effective type I error rate would rise to 0.051 or 0.06, respectively.

## 4. *p*-hacking strategies: intermediate summary

**Figure 12.**
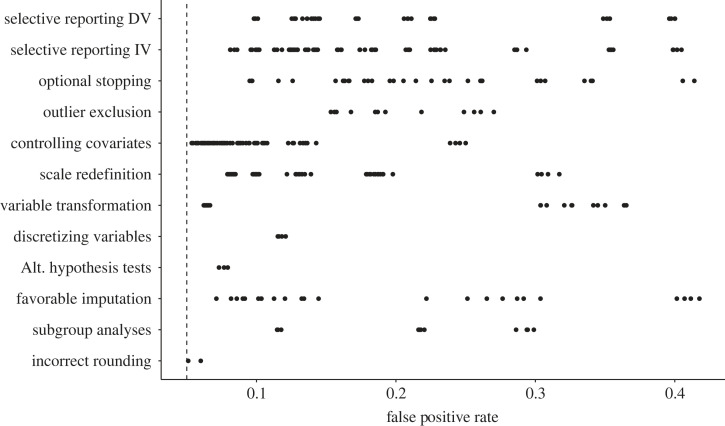
Overview of *p*-hacking severity in terms of false-positive rates for different all *p*-hacking strategies discussed in this paper.

The previous section described 12 *p*-hacking strategies and their respective impact on the type I error rates. Figure 12 compares the severity of the *p*-hacking strategies based on our simulation results.^2^ It can be seen that the variability of *p*-hacking severity is typically very high within the *p*-hacking strategies. Across all strategies, the size of the increase in error rates crucially depends on the number of tests conducted and the degree of similarity between the datasets subjected to the test. The number of tests usually corresponds to the number of ‘tweaks’ applied in a strategy, for example, the number of different (in)dependent variables, the number of outlier detection or imputation strategies, or the number of subgroups tested. Following from the principles of *α*-error accumulation, a larger number of conducted tests leads to a higher *p*-hacking severity [16]. The similarity between datasets is on the one hand determined by the correlation structure in the full dataset, for example, the correlation between different candidate dependent variables, predictors, or covariates. On the other hand, it is determined by the extent to which the *p*-hacking strategy influences the dataset. For example, in optional stopping, it matters how many new observations are added in each peek, for favourable imputation it matters what percentage of the dataset is imputed, or for selective reporting of the independent variable, it matters whether only half the data (the experimental group) or the full predictor is exchanged between tests. Generally, higher *p*-hacking severity can be expected when the tested datasets are dissimilar.

As mentioned earlier, our simulations are based on subjective plausibility assessments as well as on conventions from earlier simulation studies. This means that the variability in *p*-hacking severity depicted in figure 12 is contingent on these assumptions. If researchers are willing to exploit a *p*-hacking strategy to its limits, much higher false-positive rates are possible. We allow readers to explore these settings in our interactive Shiny app as well as through our R package for *p*-hacking simulations.

Figure 12 also makes it clear that even if only a single *p*-hacking strategy is applied to the data, false-positive rates can already increase drastically. However, judging from the fact that authors typically list more than one *p*-hacking strategy in their articles (e.g. [6,9,26]), it seems to be a consensus in the meta-scientific community that researchers often combine multiple *p*-hacking strategies when foraging their data for significant results. For this reason, the following section will investigate the combined severity of several *p*-hacking strategies by showcasing two simulated *p*-hacking ‘workflows’.

## 5. Combined application of *p*-hacking strategies

If researchers intentionally exploit degrees of freedom in their data analysis to obtain significant results, they are likely to switch to a different *p*-hacking strategy after unsuccessfully trying a few tweaks within one strategy [1]. This behaviour can be simulated as a *p*-hacking ‘workflow’ where different strategies are lined up after one another. Depending on a researcher’s level of ambition or patience, the number of strategies and the degree of aggressiveness within each strategy will differ. As with individual strategies, the *p*-hacking severity generally increases with the number of tests conducted as well as the similarity between the datasets subjected to the tests. For the combination of *p*-hacking strategies, this means that *p*-hacking severity can be expected to increase with every additional *p*-hacking strategy, as well as with the *p*-hacking severity within each of the applied strategies. However, since all strategies are applied to the same dataset, the incremental effect of each additional strategy can be smaller than the effect of the *p*-hacking strategy if applied alone.

**Figure 13.**
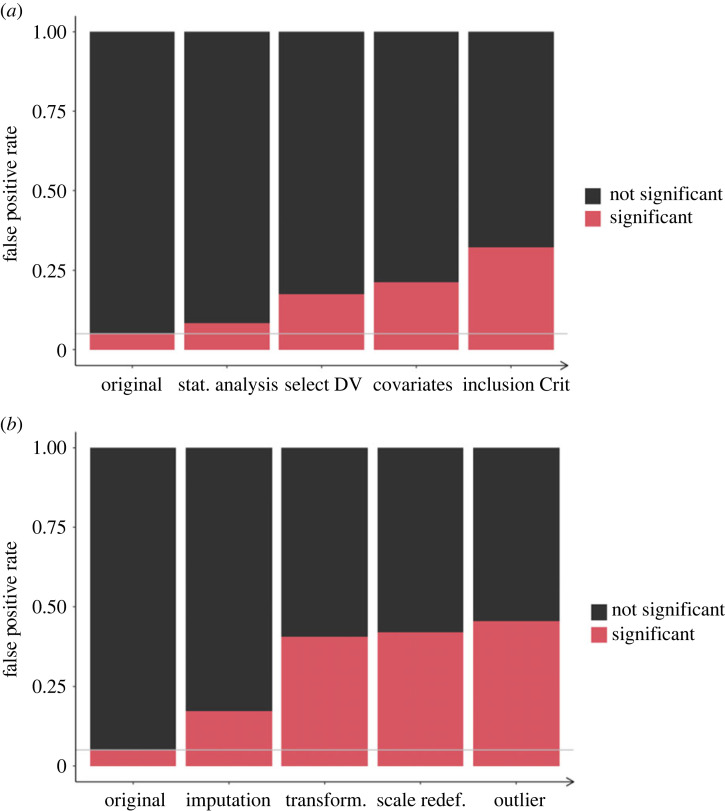
Two scenarios for *p*-hacking ‘workflows’ and their effect on false-positive rates. (*a*) *p*-hacking workflow in a *t*-test. (*b*) *p*-hacking workflow in a regression analysis. *p*-hacking strategies are applied sequentially; at each stage, incorrect rounding with a rounding level of *p* < 0.051 is applied in addition. The solid grey line shows the nominal *α*-level of 5%.

There are countless scenarios for *p*-hacking workflows that can be simulated. Here, we showcase only two scenarios using the *p*-hacking strategies described above. Figure 13 shows the simulation results. Both scenarios start with the original (planned) hypothesis test, and apply five *p*-hacking strategies successively. The figure depicts the increasing false positive rate with each additional step. We assumed a medium level of aggressiveness (compared to our earlier simulations) in each strategy, and conducted a Monte Carlo simulation with 10 000 iterations on a sample size of *N* = 100 (per group). We defined a medium level of aggressiveness as a simulation condition that would require at best moderate effort from scientists and did not show an extreme effect on error rates in our previous simulations of individual *p*-hacking strategies. Scenario (a) depicts *p*-hacking strategies applied to a *t*-test. The scenario assumes that a researcher first applies three alternative hypothesis tests (strategy 9), then tries out five dependent variables correlated at *r* = 0.6 (strategy 1), as well as three covariates correlated at *r* = 0.3 with each other and the (primary) dependent variable (strategy 5), and finally decides to restrict the sample based on three binary grouping variables (strategy 11). Scenario (b) depicts *p*-hacking strategies applied to a regression analysis. The scenario assumes that a researcher first imputes missing data (10% of the total sample) using five random imputation methods (strategy 10). If this does not yield a significant result, missing cases are deleted, and the researcher proceeds to repeating the regression analysis while applying different transformations to the predictor and outcome variable (strategy 7). Then, the dependent variable (psychological scale consisting of five items) is redefined by successively deleting up to three items from the score (strategy 6), and finally, three random outlier detection methods are applied, and the test is repeated without the outliers (strategy 4). In both strategies, we assumed that researchers apply incorrect rounding (strategy 12) at every stage of the *p*-hacking process. In the *t*-test scenario, this was responsible for 2.9% of false-positive results, and in the case of the regression scenario for 2.5%.

For the regression scenario, the combination of *p*-hacking strategies indeed led to a higher rate of false-positive results than any single *p*-hacking strategy. However, it is also visible that the incremental effect of each additional strategy decreases. For example, if used as a singular strategy at the same level of aggressiveness, the outlier exclusion strategy would have led to a type I error rate of 15%, that is, an increase of 10 percentage points compared to the original *α*-level (figure 4). By contrast, in the combined scenario, it only led to an increase of 3.5 percentage points. False-positive rates in the *t*-test scenario remained below the maximum false-positive rates observed in the simulations of individual strategies. However, this is due to the fact that none of the strategies was fully exploited.

## 6. Ambitious *p*-hacking: the influence of reporting strategies

Since *p*-hacking is based on selecting analyses to report among multiple tests, it is often interpreted as self-imposed publication bias [8]. Most authors assume that researchers *p*-hack until they obtain a significant result, then stop conducting additional tests, discard the series of initial non-significant results, and report the one significant outcome (e.g. [9]). However, this view has been challenged by other authors who suggested that researchers may in fact conduct hypothesis tests in batches [8,27]. For example, if researchers have collected a set of *k* candidate dependent variables, the authors assume that researchers would calculate all *k* tests and subsequently report the smallest *p*-value (independent of statistical significance) or the smallest significant *p*-value. This behaviour has sometimes been labelled *ambitious* *p*-hacking [89].

Based on our simulation studies, we argue that ambitious *p*-hacking should not be used to distinguish between *p*-hacking strategies. For example, outlier exclusion and *ambitious* outlier exclusion should be considered the same *p*-hacking strategy. Our reasoning is as follows: every *p*-hacking strategy presented above can be conducted at different levels of aggressiveness. Researchers who are sufficiently dedicated or unscrupulous may be willing to conduct many hypothesis tests within each of the described *p*-hacking strategies to find a significant result. Whether they find this significant result earlier or later in the process is mostly a matter of luck.^3^ The resulting false-positive rate therefore depends entirely on how many tests researchers would have been willing to conduct. As soon as the researchers are able to obtain a single significant result, the *p*-hacking procedure was ‘successful’. It does not matter whether this significant result occurred earlier or later, and it does not matter whether more significant results would have been obtained if the researchers had continued to *p*-hack until they reached the maximum number of tweaks they were willing to make. The operating characteristic of the statistical procedure, that is, the false-positive rate, depends on the hypothetical number of tests that could have been conducted [90]. Therefore, ambitious *p*-hacking does not change the false-positive rate of the procedure.

The aspect of the procedure that changes with ambitious *p*-hacking is the selection of the reported *p*-value. Here, we call the selection rules the *reporting strategy*. Based on simulation studies by van Aert *et al.* [27] and [8] we distinguish between two reporting strategies in ambitious *p*-hacking: reporting the *smallest* *p*-value and reporting the *smallest significant* *p*-value. When reporting the smallest *p*-value, researchers always report the smallest *p*-value out of a batch of hypothesis tests independent of statistical significance. When reporting the smallest significant *p*-value, researchers report the smallest *p*-value among all significant *p*-values in a batch, but revert to the *p*-value of the original (planned) test if none of the computed tests achieved statistical significance. Theoretically, many other reporting strategies are possible. For example, researchers may only choose to report a smaller *p*-value if it is considerably smaller than their original *p*-value, or researchers may always report the smallest *p*-value *unless* they obtained a significant result. However, for the purpose of illustration, two reporting strategies are sufficient.

**Figure 14.**
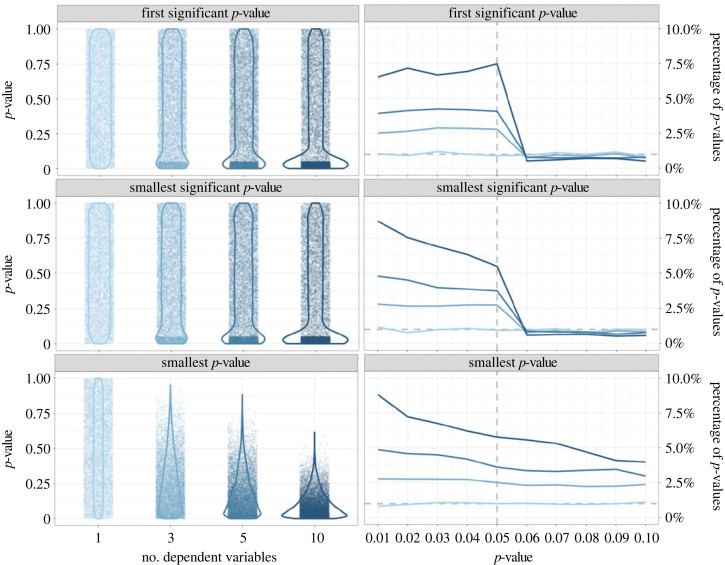
Distribution of *p*-values under three different reporting strategies based on the *p*-hacking strategy ‘selective reporting of the dependent variable’: reporting the first significant, smallest significant or smallest *p*-value out of a batch of tests. Left side shows full *p*-value distribution, right side shows *p*-curves [34]. One dependent variable indicates no *p*-hacking, *p*-hacking aggressiveness increases with the number of dependent variables. Simulation with *N* = 100, *ρ*_*DV*_ = 0.3.

Although reporting strategies do not affect the false-positive rate of the procedure, they do affect the *p*-value distribution across multiple *p*-hacked hypothesis tests or studies. Figure 14 illustrates this using the *p*-hacking strategy ‘selective reporting of the dependent variable’ (strategy 1; for other strategies see our electronic supplementary material, online appendix). As can be seen from the panels on the left side of the figure, more aggressive *p*-hacking leads to a higher concentration of *p*-values below the significance threshold. Without *p*-hacking (here: one dependent variable), the *p*-value distribution is uniform for a null-effect. With increasing *p*-hacking aggressiveness (here: 3, 5 or 10 dependent variables), the *p*-value distribution concentrates around smaller *p*-values. For any given level of *p*-hacking aggressiveness, the proportion of significant *p*-values is the same across reporting strategies. However, the reporting strategy influences the overall shape of the *p*-value distribution: if the smallest significant *p*-value is reported, the overall *p*-value distribution is very similar to the distribution arising from stopping after the first significant *p*-value depicted in the upper panel. If the smallest *p*-value is reported independent of statistical significance, the overall *p*-value distribution becomes skewed towards smaller *p*-values. The panels on the right side of figure 14 zoom in on small *p*-values using the *p*-curve method [34]. Here, the range from *p* = 0 to *p* = 0.1 is divided into 10 equally sized intervals, and the percentage of simulated *p*-values falling into the interval is recorded. Without *p*-hacking, each interval should theoretically contain 1% of *p*-values (see horizontal dashed line and lightest blue line). Depending on the reporting strategy, the *p*-curve takes different shapes: if the first or smallest significant *p*-value is selected, the *p*-curve displays a clear break point around the significance level. If the smallest *p*-value is selected, the *p*-curve shows a more continuous decrease. Additionally, if the smallest or smallest significant *p*-value is reported, the *p*-curve is right skewed for values below the significance level. In our view, it is beneficial to differentiate between *p*-hacking and reporting strategies for several reasons. First, it sharpens the terminology surrounding *p*-hacking by clarifying what procedures should be viewed as separate *p*-hacking strategies. Second, it makes clear that the rate of false-positive results only depends on the *p*-hacking strategy, but not on the selection of the reported significant *p*-value. Third, it supports the interpretation of *p*-curves in the presence of *p*-hacking. We therefore hope that the distinct terminology will be used in future research.

## 7. Evaluating potential solutions to the problem of *p*-hacking

In the past sections, we have described the influence of different *p*-hacking strategies on the type I error rates as well as on the distribution of *p*-values using simulation studies and a classification of different *p*-hacking strategies. In the following, we want to use these results to evaluate the potential of several solutions that have been proposed to counter the issue *p*-hacking and increase research credibility.

### 7.1. Larger sample sizes

In the wake of the replication crisis, many authors have called for larger sample sizes (e.g. [46,91,92]). As a reaction, several academic journals introduced recommendations for minimum sample sizes or made power analyses a prerequisite for publication. Larger sample sizes have many advantages. Most importantly, they increase statistical power, that is, the probability of finding an existing effect, and lead to a higher precision of parameter estimates. However, our simulations show that in the absence of an effect larger sample sizes alone rarely protect against *p*-hacking. If a researcher is willing to hack a dataset, they will likely be successful despite the large sample size. This is important to know because it suggests that even in an environment with high statistical power, researchers need to look out for signs of questionable research practices and cannot rely on a ‘fail-proof’ N.

In practice, requirements for large sample sizes may affect incentives for *p*-hacking in several ways. Due to resource constraints, re-conducting large studies for the sake of obtaining significant results in a second sample may become impossible for many researchers. On the one hand, this could encourage researchers to report null results. This might be due to the fact that researchers believe that null results based on large sample sizes are more indicative of a true null effect, or because they believe that throwing away large amounts of data would have a more detrimental effect on their career than publishing null results (perhaps contradicting their own earlier findings). On the other hand, not being able to re-conduct a study may increase researchers’ willingness to *p*-hack the available dataset more aggressively since trying out more statistical tests would imply less effort than collecting additional data in publishable quantities. Which one of these motivations prevails is difficult to say in advance and could pose an interesting question for further empirical investigation.

The simulations presented in this paper only investigated *p*-hacking in the absence of an effect. However, *p*-hacking may also occur if an effect is present in the population. In the presence of an effect, larger sample sizes increase the probability of obtaining a significant result. With a significant result at hand, researchers will typically see no more need to *p*-hack (but see our earlier discussion of ambitious *p*-hacking for an alternative viewpoint). Therefore, even though large sample sizes are usually not effective at reducing *p*-hacking effects when *p*-hacking takes place, they may have a preventative function by decreasing incentives for *p*-hacking in the presence of an effect.

### 7.2. Redefine statistical significance

Faced with the high proportion of false-positive results in the social science literature, a group of 72 researchers proposed to lower the default threshold for statistical significance from *α* = 0.05 to *α* = 0.005 [45]. In the absence of questionable research practices this change in standards decreases the maximum false-positive rate by a factor of 10. Is the same true in the presence of *p*-hacking?

**Figure 15.**
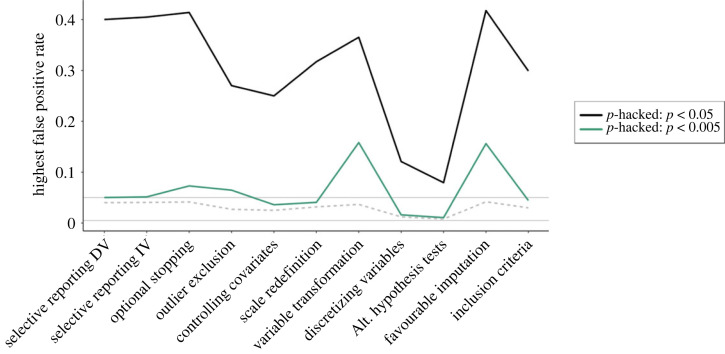
Highest false-positive rates based on simulation conditions reported earlier for all *p*-hacking strategies for significance levels of *α* = 0.05 and *α* = 0.005. Solid grey lines indicate nominal *α* levels, dashed grey line indicates 10-fold reduction in false-positive rates compared to ‘standard’ *p*-hacking for *p* < 0.05.

Figure 15 shows the highest false-positive rates obtained for each *p*-hacking strategy in the simulations reported earlier (black line) together with the highest false-positive rates obtained from *p*-hacking for *p* < 0.005 (green line). As such, the figure represents a worst-case scenario: it shows the highest false-positive rates that can be observed with ‘reasonably’ aggressive *p*-hacking (as defined in our simulations) for both significance thresholds. As can be seen, the highest observed false-positive rates drop substantively after redefining the *α*-level. But do they decrease at the same rate as would be expected in an ideal scenario without questionable research practices? The dashed grey line in the figure represents a 10-fold decrease compared to the false-positive rates obtained with a threshold of *α* = 0.05. If decreasing the significance level led to the same decrease in false-positive rates in the absence and presence of *p*-hacking, false-positive rates in the presence of *p*-hacking should decrease from the height of the solid black line to the height of the dashed grey line. As can be seen, false-positive rates for *p*-hacking at *p* < 0.005 are higher than the dashed grey line. This indicates that *p*-hacking can partially reverse the beneficial effects of lowering the significance threshold on type I error rates. However, even in the presence of strong *p*-hacking, as depicted in the figure, false-positive rates for the reduced significance level are typically much lower than for *p*-hacking at the 0.05-level. Thus, while the beneficial effect of redefining statistical significance may not be as strong in the presence as in the absence of *p*-hacking, it can still substantively decrease the success rate of *p*-hacking, and therefore potentially render it less attractive.

### 7.3. A *δ* for every *p*: shifting the focus to effect sizes

As a response to ubiquitous criticism of null hypothesis significance testing, several groups of methodologists have called for abandoning *p*-values in favour of alternative statistical approaches. One of these approaches centres around replacing frequentist hypothesis testing with effect size estimation [93]. Effect sizes are measures of quantities of interest such as mean differences, correlations or odds ratios. Commonly, effect sizes are reported as unit-free standardized measures that allow comparisons of effects across different experimental contexts. Examples for standardized effect size measures include Cohen’s *δ*, Pearson’s correlation coefficient *ρ*, or the partial *η*^2^ for ANOVAs [94].

**Figure 16.**
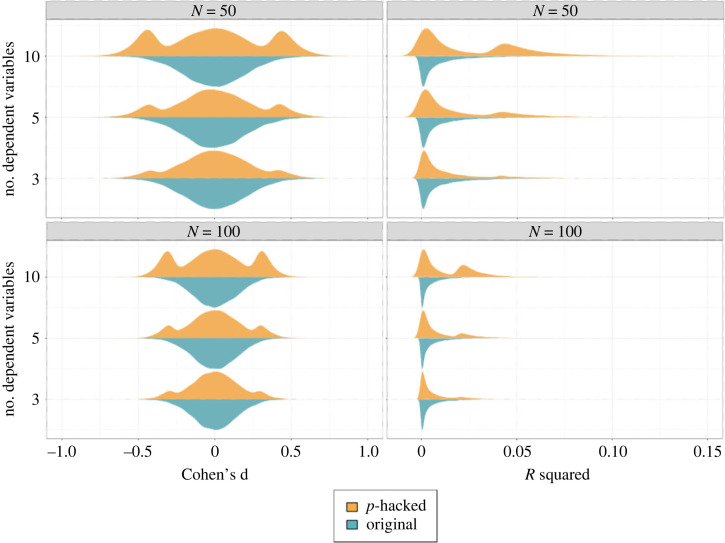
Distribution of effect sizes for *p*-hacked and original (non-*p*-hacked) analyses for two sample sizes *N* ∈ {50, 300}. *p*-hacking is based on selective reporting of the dependent variable; first significant *p*-value is reported. Simulation with *ρ*_*DV*_ = 0.

As noted at the beginning of this paper, fishing for publishable results can occur in any statistical framework, with effect size based inference being no exception. However, different statistical targets are susceptible to different hacking strategies [23]. The simulations conducted in this paper do not encompass dedicated ‘effect size hacking’ strategies, and it is out of the scope of this manuscript to discuss potential differences between *p*-hacking and effect size hacking strategies in detail. However, we can evaluate the effect of *p*-hacking strategies as described above on the distribution of effect sizes. Figure 16 illustrates the influence of selecting among *k* ∈ {3, 5, 10} uncorrelated dependent variables in a two-sided *t*-test on effect sizes, if the first significant result is reported. The panels on the left side show the distributions of Cohen’s *d* with and without *p*-hacking for two sample sizes, *N* ∈ {50, 300}. Without *p*-hacking, effect sizes are distributed in a symmetric and unimodal distribution around zero that decreases in width for larger sample sizes. With increasingly aggressive *p*-hacking, two additional peaks appear at non-zero effect sizes. These belong to hypothesis tests where *p*-hacking was successful. As can be seen on the right side of figure 16, the effect of *p*-hacking on the coefficient of determination R^2^ shows a similar trend, where original *p*-values peak around zero and effect sizes are biased towards larger values if *p*-hacking took place. With larger sample sizes, the observed bias shrinks, and effect sizes move towards zero again. Note that the exact distribution of effect sizes depends on the reporting strategy. Effect size distributions for other *p*-hacking and reporting strategies can be found in our electronic supplementary material, online appendix.

To summarize, inflated effect sizes are a mathematical consequence of *p*-hacking that is more pronounced in small samples. Hence, effect sizes are overestimated under the presence of *p*-hacking. For increasing sample sizes and a true null effect, the *p*-hacked effect sizes move towards zero, such that in the limits the influence of *p*-hacking disappears. Therefore, reporting effect size measures in addition to significance testing results may help in situations where sample sizes are large, because it becomes clear that the effect size is minuscule despite the significant test result. However, especially in the presence of a significant hypothesis testing result, it seems likely that researchers overinterpret the practical relevance even for minuscule effects. This is also the hidden danger of completely replacing *p*-values with effect size measures: although the outcome hacking strategies in our simulations were not ‘optimized’ for effect size hacking, they show that it is technically possible to inflate effect sizes by using techniques similar to *p*-hacking. Such inflated effect sizes can easily be interpreted as ‘practically’ significant [95], even if they are close to zero. Thus, switching from *p*-values to effect size estimation provides little protection from an inflation of false-positive claims in the literature.

### 7.4. A *B* for every *p*: shifting the focus to Bayes factors

While some researchers promote focusing on effect sizes, others advocate for changing the statistical hypothesis testing framework. Frequentist hypothesis testing has been criticized for numerous issues, its fallibility for *p*-hacking being only one of them [90]. Therefore, a growing number of methodologists argue in favour of switching to the Bayesian statistical framework for data analysis to improve statistical inferences [23,96]. The quantity of interest in Bayesian hypothesis testing is the Bayes factor. It is a relative measure of evidence and compares the likelihood of observing the data under the null and alternative hypothesis. Bayes factors (BF_10_) larger than one indicate evidence in favour of the alternative hypothesis, Bayes factors smaller than one indicate evidence in favour of the null hypothesis [97]. While proponents view the Bayes factor as a continuous quantification of evidence that resists all-or-nothing decisions, critics have argued that in practice, Bayes factors crossing certain thresholds are viewed as good-enough evidence to make a decision about the tested hypotheses [98,99]. Since decisive evidence is usually preferable for publication, this suggests that incentives for outcome hacking may persist despite a switch to Bayesian inference.

**Figure 17.**
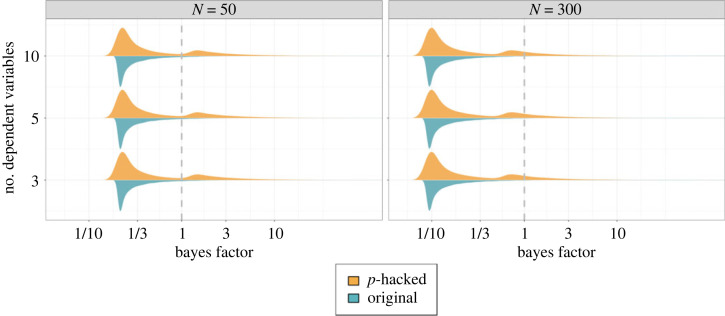
Distribution of Bayes factors (BF_10_) for *p*-hacked and original (non-*p*-hacked) analyses for two sample sizes *N* ∈ {50, 100}. *p*-hacking is based on selective reporting of the dependent variable; first significant *p*-value is reported. Simulation with *ρ*_*DV*_ = 0; Bayes factors computed with default priors following Morey & Rouder [102].

As with effect sizes, it is outside the scope of this article to discuss potential dedicated Bayes factor hacking strategies. However, we can evaluate the influence of *p*-hacking on Bayes factors in the light of our simulation results, and we can evaluate whether a less radical proposal—to report a Bayes factor for every *p*-value [100]—may be beneficial. In an earlier simulation study, Simonsohn [101] argued that *p*-hacking strategies can also affect Bayesian inference. Our own results generally support this notion. Figure 17 displays the distribution of Bayes factors under the null hypothesis in a two-sided *t*-test under different levels of *p*-hacking severity and for two sample sizes, *N* ∈ {50, 300}. We assume that the first significant *p*-value is reported. It can be seen that the original Bayes factors concentrate around 1/5 or 1/10 for the two sample size conditions, respectively, if no *p*-hacking is applied, thus correctly indicating evidence for the null hypothesis. For *p*-hacked tests in the small sample size condition, *p*-hacked Bayes factors tend to show weak evidence for the alternative hypothesis. In the large sample size condition, even *p*-hacked Bayes factors largely show (albeit weak) evidence in favour of the null. Note that the exact value of the Bayes factor depends on the specification of the prior distribution. The Bayes factors displayed in figure 17 use a default JZS prior distribution that is implemented in a commonly used software package for Bayesian hypothesis testing [102]. This prior is considered a relatively conservative choice and tends to pull the Bayes factor towards the null hypothesis in the presence of small effect sizes and small sample sizes [96]. For more informed priors, the Bayes factor distributions could therefore be more favourable towards the alternative hypothesis.

The exact distribution of Bayes factors also depends on the reporting strategy. Importantly, under the ‘report the smallest *p*-value’ strategy, the Bayes factor distribution is no longer bimodal under the presence of *p*-hacking, but it is still biased towards larger Bayes factors. Bayes factor distributions for all reporting strategies can be found in our electronic supplementary material, online appendix.

Taken together, our results indicate that Bayes factors are not immune to *p*-hacking, in that they will overstate the evidence for the alternative hypothesis even for large sample sizes if *p*-hacking was successful. However, importantly, for large sample sizes Bayes factors can eventually correctly indicate evidence for the null hypothesis even in the presence of *p*-hacking. If researchers report ‘a *B* for every *p*’ ([100], p. 216), this means that under these circumstances inconsistencies between frequentist and Bayesian hypothesis testing results may arise. If confronted with these inconsistencies, we believe that researchers may interpret findings with greater care. Therefore, the suggestion to report ‘a B for every p’ appears to be useful advice even for researchers preferring the frequentist approach for epistemological reasons. However, it is important to stress again that our simulations only used default Bayes factors and did not encompass dedicated ‘*B*-hacking’ strategies that are targeted at lifting Bayes factors across specific thresholds [23]. Additionally, our simulations only examined outcome hacking in favour of the alternative hypothesis, whereas researchers may also be motivated to find strong evidence for the null hypothesis when using Bayes factors. Therefore, *B*-hacking may be more effective in practice than our simulation results suggest.

### 7.5. Correct for *p*-hacking

As long as incentives in science do not change, many researchers argue that *p*-hacking will remain a problem [1]. Therefore, analogous to publication bias correction methods, reliable *p*-hacking detection and correction methods could help scientists to interpret potentially *p*-hacked findings with more caution and correct for biased estimates. In the past, several methods have been applied for *p*-hacking detection, such as *p*-curve [12,34], the Caliper test [61,103], Fisher’s method [104] or excess test statistics [13].

In our view, current *p*-hacking detection and correction methods suffer from two shortcomings. First, it is impossible to make inferences about *p*-hacking in a single study. All *p*-hacking correction methods developed so far rely on the close investigation of an empirical *p*-value distribution. This requires the existence of multiple *p*-values stemming from the same population distribution (e.g. a null effect). However, especially for novel findings, no comparable findings might exist, and therefore also no empirical distribution. Moreover, even if many hypothesis tests exist, *p*-hacking detection methods can only make inferences about whether *p*-hacking is present or absent across all tests. Therefore, finding evidence for *p*-hacking in a body of literature may decrease researchers’ confidence in a research field, but it does not imply that any specific study result was *p*-hacked. Second, *p*-hacking detection and correction methods do not take the diversity of *p*-hacking and reporting strategies into account. As we discussed earlier, different reporting strategies can lead to different *p*-value distributions across multiple hypothesis tests (figure 14). Not all of these reporting strategies lead to a right-skewed distribution of *p*-values below *p* = 0.05, as is assumed by many current *p*-hacking detection methods. This indicates that specific types of *p*-hacking would not be detected by current methods. Moreover, in practice, *p*-hacking in a research field can hardly be assumed to be an all-or-none issue. It is unlikely that all researchers *p*-hack their data, and even if they do, they are unlikely to use the same *p*-hacking strategies at the same level of aggressiveness combined with the same reporting strategies. Therefore, it must be assumed that the resulting distribution of *p*-values emerges as a mixture of different *p*-hacking strategies, which makes correcting for a bias caused by *p*-hacking difficult, if not nearly impossible.

### 7.6. Preregistration and registered reports

At the basis of *p*-hacking lies the exploitation of undisclosed analytic flexibility in scientific data analysis. In the past years, it has been a cornerstone of the scientific reform movement to promote publication practices that require researchers to specify planned analyses and thereby limit analytic flexibility. Among these practices, the main focus has been on preregistration and registered reports [105,106].

The idea of preregistration stems from medical research where it was introduced in the form of clinical trial registrations in the 1960s [107]. A preregistration is a time-stamped document in which researchers describe their hypotheses, methods, and analyses prior to data collection and/or data analysis [108]. Ideally, a preregistration provides a detailed overview of all planned analysis steps that can be referenced in a publication to clearly distinguish confirmatory from exploratory analyses [20]. However, analysis plans laid out in preregistrations are often vague, analyses are quietly changed or incompletely reported in publications, and sometimes preregistrations are even written retrospectively [109–111]. All these aspects clearly limit the value of preregistrations in practice and spawned the development of registered reports.

Registered reports were first introduced in the neuroscience journal *Cortex* in 2015 [112]. They seamlessly incorporate preregistration in the publication process: in a first stage, authors submit the information typically contained in a preregistration to a journal in the form of a scientific report. Based on peer reviews, this stage 1 report can obtain in principle acceptance, meaning that the journal commits to publishing the study results independently of whether they support the hypotheses or not. Once the authors have collected their data and conducted all preregistered analyses, they can submit a full scientific paper to the journal that will be published provided that the authors followed their outlaid research agenda [113]. Compared to preregistrations, registered reports have the advantage that the registration of planned analyses is guaranteed to occur prior to data collection. Additionally, pre-data preregistration quality and post-data preregistration compliance can be easily monitored through peer review. Therefore, registered reports are currently considered the highest quality standard for confirmatory research [114].

Whether preregistrations and registered reports can suppress *p*-hacking crucially depends on whether they can effectively constrain the number of analysis options. Our simulation results demonstrate that vague descriptions of analyses that do not consider all degrees of freedom in data preprocessing and analysis pipelines leave the door wide open to *p*-hacking. For example, a preregistration may describe a planned regression analysis, but fail to report outlier exclusion and missing value handling procedures. This means that a researcher is now technically free to use the corresponding *p*-hacking strategies without being detected in the process. Essentially, as long as a single aspect of an analysis remains undefined, *p*-hacking remains possible. In registered reports, such loopholes may be detected during the initial peer review phase and can be fixed by researchers prior to data collection. However, verbal descriptions of analyses are inherently vague. A stricter analysis registration format would therefore require the *a priori* specification of the entire data analysis script [115,116]. This could, for example, be demanded in the context of peer review in a registered report process.

No matter whether the preregistration comes in the form of verbal descriptions or code, it is our hope that the compendium of *p*-hacking strategies presented in this paper will sharpen the eyes of potential reviewers towards remaining degrees of freedom and contribute to the improvement of already existing preregistration templates (e.g. [108]). In practice, it may be difficult for researchers to specify all aspects of their analyses in advance, such that adjustments or corrections are necessary [117,118]. In these cases, it is vital that researchers are transparent and demonstrate the sensitivity of their conclusions to the post hoc decisions. In this way, analytic flexibility that was not eliminated *a priori* can be made visible and selective reporting can be avoided.

## 8. Discussion and conclusion

In the wake of the replication crisis, detecting and preventing questionable research practices has become a major concern for many researchers. Among all questionable research practices, *p*-hacking has been the centre of attention. However, definitions of *p*-hacking have been surprisingly vague, and the severity of different *p*-hacking strategies has never been explored in detail. In this paper, we compiled a list of 12 *p*-hacking strategies targeted at common statistical testing scenarios that have been mentioned in the literature. We investigated the severity of each of these strategies for different levels of *p*-hacking aggressiveness using simulation studies. Then, we used the results of these simulations to provide a preliminary evaluation of the effect of several commonly suggested measures for improving statistical inferences and the trustworthiness of research results.

With regard to the different *p*-hacking strategies, we found that even with a single strategy, false-positive rates can typically be raised to at least 30% from the typical 5% threshold with ‘reasonable effort’, that is, without assuming that researchers automate data mining procedures. Interestingly, some *p*-hacking strategies that have received comparatively little attention in the existing literature, such as variable transformation (strategy 7) or favourable imputation (strategy 10), showed the most severe effects on error rates in our simulations. Apart from the aggressiveness of *p*-hacking itself, our simulations showed that across all strategies, the severity of *p*-hacking also depends on the environment in which *p*-hacking takes place, for example, the correlation structure in the data. We believe that this is an important factor to consider in future investigations of *p*-hacking severity as well as in the context of the development of *p*-hacking detection and correction methods.

In our simulation framework, we demonstrated that it can be useful to separate *p*-hacking strategies from reporting strategies. In the literature, there has been some disagreement on whether researchers report the first significant *p*-value or conduct several tests at the same time and report, for example, the smallest or smallest significant *p*-value [8,9,27]. However, these reporting strategies have always been viewed as integral to *p*-hacking strategies. By viewing *p*-hacking as a group of strategies to obtain statistical significance, it becomes clear that reporting strategies should be viewed as separate from *p*-hacking strategies. False-positive rates are purely determined by the aggressiveness of the *p*-hacking strategy employed, but reporting strategies have an additional effect on the resulting *p*-value distribution. In our view, this means that authors developing new methods to investigate *p*-hacking should always be clear on whether they target *p*-hacking or reporting strategies.

With regard to potential solutions to the problem of *p*-hacking, we found that reporting Bayes factors in addition to *p*-values may be the best option to enable a critical evaluation of research results. For large sample sizes, reporting effect size estimates may also lead readers to question significant results, since these will typically be paired with small effect size estimates in the case of *p*-hacking. Interestingly, larger sample sizes alone are not enough to reduce the ‘success rate’ of *p*-hacking attempts. Reducing the threshold for statistical significance, however, also leads to a substantive decline in false-positive rates. Publication models aiming at preventing *p*-hacking, specifically preregistration and registered reports, can theoretically be effective if they succeed at eliminating analytic flexibility. However, based on our simulations, it became clear that even small loopholes in preregistrations, that is, degrees of freedom that are not fixed, can render preregistrations ineffective towards severe *p*-hacking with the remaining strategies. We also considered *p*-hacking detection and correction methods as a potential solution for the case that *p*-hacking cannot be successfully prevented. However, due to the multitude of different strategies and the heterogeneity of research scenarios, it seems unlikely that a reliable detection mechanism can be developed in the near future.

It is important to note that all simulation results regarding *p*-hacking severity in this paper depend on our specific implementation of *p*-hacking strategies, as well as on our subjective assessment of plausible *p*-hacking aggressiveness. While there are several surveys that allow inferences about the prevalence of certain *p*-hacking strategies (e.g. [4,5]), none of these surveys has investigated the aggressiveness with which researchers employ these strategies. It is also unclear how many strategies researchers would typically use at a time, or whether certain fields of research are more fallible to some strategies than to others. These are still open empirical questions that we hope will be investigated in the future. Our simulation conditions are therefore merely representations of our individual assessments of plausible effort. Whether or not they are realistic may be determined by future empirical studies. We encourage readers who disagree with our simulation settings to explore other conditions using our Shiny app or our R package, and to adapt the code that we provided (https://github.com/astefan1/phacking_compendium). We believe that starting a debate about what *p*-hacking realistically looks like in practice will eventually improve our approaches towards *p*-hacking prevention.

Throughout the whole article, we relied on Monte Carlo simulations to assess the severity of *p*-hacking effects. Simulation studies are a straightforward and flexible approach to determine the operational characteristics of statistical procedures. However, at least some of the *p*-hacking strategies presented in this paper can also be mathematically modelled. Exact mathematical descriptions of *p*-hacking strategies have the advantage that the properties of *p*-value distributions can be theoretically derived without estimation errors, and costly computations can be avoided. Additionally, they may contribute to the refinement of selection models of publication bias by making it possible to include information about *p*-hacking [28,119]. Future research should therefore be directed at developing mathematical models of *p*-hacking strategies.

Gazing into the abyss of *p*-hacking may be daunting, but in our view, it is a prerequisite for developing effective countermeasures. The increased awareness of *p*-hacking in general has already led to many improvements in statistics education, publication practices, and research design. By framing *p*-hacking as a compound of strategies, we hope that actions can become even more targeted in the future. Our compendium and simulation of *p*-hacking strategies can be viewed as a first step in this direction.

## Data Availability

Data and relevant code for this research work are stored in GitHub: https://github.com/astefan1/phacking_compendium and have been archived within the Zenodo repository: https://doi.org/10.5281/zenodo.7510292 [120]. The data are provided in electronic supplementary material [121].

## Footnotes

1 The term *procrustean* stems from Greek mythology, where Procrustes, an infamous robber, made his victims fit the length of his iron bed by stretching them or cutting off their legs.

2 In case of incorrect rounding, the depicted false-positive rates are the analytic examples provided in the text.

3 The search for significant results could theoretically be optimized, but here we assume that researchers do not automate or optimize any part of the process as this would indicate malicious intent.
